# Severe ovarian hyperstimulation syndrome following sole gonadotropin-releasing hormone (GnRH) agonist trigger: a case series and literature review

**DOI:** 10.1007/s00404-024-07740-7

**Published:** 2024-09-20

**Authors:** Roza Berkovitz-Shperling, Nivin Samara, Reut Meir, Omri Dominsky, Foad Azem, Ido Feferkorn

**Affiliations:** 1 Department of Obstetrics and Gynecology, Lis Hospital for Women’s Health, Tel Aviv. Sourasky Medical Center, affiliated to the Faculty of Medicine, Tel Aviv University, 6423906 Tel Aviv, Israel; 2 Department of Obstetrics and Gynecology, Shaare Zedek Medical Center, 6 Weizman St., Affiliated to Faculty of Medicine, The Hebrew University of Jerusalem, Jerusalem, Israel

**Keywords:** GnRH antagonist, Ovarian hyperstimulation syndrome, GnRH agonist triggering

## Abstract

**Objective:**

The aim of this study was to report three cases of early severe ovarian hyperstimulation syndrome (OHSS) in patients undergoing a GnRH antagonist protocol triggered with GnRH agonist (GnRH-a), leading to hospitalization and the need for peritoneal drainage. Additionally, a review of the existing literature on this topic is provided.

**Design:**

This is a retrospective case series and a literature review.

**Setting:**

This study was conducted at obstetrics and gynecology department of tertiary academic referral hospitals, Israel.

**Participants:**

This study included three patients presented with severe OHSS symptoms, including abdominal distension, ascites, and hemoconcentration.

**Main outcome measures:**

The main focus of the treatment was to address the symptoms and prevent any further complications. The outcome was the complete recovery of the patients.

**Results:**

The presented cases detail instances of severe OHSS following oocyte retrieval, utilizing GnRH-a for triggering. Case 1 involved a 33-year-old patient with a history of polycystic ovary syndrome (PCOS), Case 2 featured a 22-year-old patient with familial adenomatous polyposis (FAP), and Case 3 included a 41-year-old patient with a history of depressive disorder. All patients receiving supportive care, including infusions and medications, exhibited gradual improvement during hospitalization, with complete resolution observed during the 20-day post-hospitalization check-up.

**Conclusions:**

These three cases highlight the occurrence of severe early OHSS following a GnRH antagonist protocol triggered with GnRH-a in the absence of human chorionic gonadotropin (hCG) administration for trigger or luteal-phase support. Clinicians must be aware that a GnRH-a trigger followed by a freeze-all approach does not guarantee the complete elimination of OHSS in all patients.

## What is already known on this topic

**Table:** 

OHSS is a complication associated with IVF, potentially leading to hospitalization and, in severe instances, even patient mortality. A widely adopted strategy to mitigate OHSS risk involves utilizing GnRH-a as a trigger for the final maturation of oocytes. However, a limited number of case reports have documented instances of OHSS occurring after the administration of GnRH-a.
**What this study adds t**hree cases of severe OHSS following GnRH-a administration are presented, highlighting the condition's severity, treatment strategies, and resolution outcomes. This is further supplemented by a thorough review of the relevant literature
**How this study might affect research, practice, or policy** - This study summarizes the existing literature on the topic, aiming to raise awareness of this rare phenomenon. By addressing uncommon diagnoses, it encourages a broader perspective on this rare condition, encompassing considerations for diagnosis, treatment, and resolution.

## Introduction

Ovarian hyperstimulation syndrome (OHSS), as classified by Golan and Navot [1, 2], is an iatrogenic complication stemming from fertility treatment [3], characterized by an exaggerated response to controlled ovarian stimulation (COS) that causes protein-rich fluid to shift from the intravascular space to the third space [4].

Historically, about one-third of in vitro fertilization (IVF) cycles showed mild OHSS and severe OHSS cases were reported in 0.5–6% of IVF cycles [5–7]. Risk factors for OHSS primarily correlate with the number of developing follicles and retrieved oocytes, with notably heightened risk when more than 15 oocytes are collected [8].

The clinical presentation of OHSS varies based on fluid translocation intensity and consequent blood volume reduction. Symptoms range from mild abdominal swelling due to enlarged ovaries or abdominal fluid accumulation to severe outcomes such as kidney failure or mortality [9]. Hemodynamic alterations leading to hemoconcentration contribute to compromised organ perfusion thromboembolism and affect vital organs like the kidneys, heart, and brain, accentuating OHSS severity [4, 10]. OHSS can be defined as early OHSS (beginning 1–7 days after Human chorionic gonadotrophin (hCG) trigger and usually resolving with menses) or late (typically beginning 9–10 days after hCG trigger in response to the rising hCG of pregnancy) [11].

The presence of Human chorionic gonadotropin (hCG), which is commonly used for ovulation induction due to its extended half-life and strong luteinizing hormone (LH) receptor affinity [12], fosters OHSS development by exerting a prolonged luteotropic effect, prompting excessive vascular endothelial growth factor (VEGF) production [13–15], thus contributing to heightened vascular permeability [16–18]. In cases of excessive ovarian response with high OHSS risk, triggering final follicular maturation with GnRH-a [19], combined with a freeze-all approach, is a prevalent strategy [6, 20]. Although initially believed to eliminate OHSS incidence, [14, 21, 22] this is likely not the case, and it was cautioned that such a conclusion is premature [10, 12–14, 16–18, 23–29]. Reported OHSS cases after GnRH-a triggering [30, 31] were questioned, as some involved simultaneous hCG use (as luteal support) or pregnancy (intrauterine or ectopic) [32], where naturally occurring hCG could contribute to OHSS occurrence. Hence, the role of GnRH-a alone in causing OHSS remained debated [8, 28]. A recent study by Fernandez et al. [10] explored 77 high responders who underwent GnRH-a triggering and found that 17 patients (22%) experienced mild early and late OHSS symptoms, including abdominal discomfort, enlarged ovaries, nausea, and minor ascites. Notably, none of these patients developed severe early OHSS, which is the more clinically relevant adverse effect.

While most cases report on OHSS following GnRH-a trigger focus on mild to moderate cases, here we report on three cases where GnRH-a trigger led to early-onset severe OHSS necessitating hospitalization and peritoneal drainage.

## Material and methods

A comprehensive literature search was conducted using electronic databases such as PubMed, Embase, and Google Scholar. Inclusion criteria encompassed all studies, focusing on OHSS cases occurring after the administration of GnRH agonists alone. Relevant keywords and MeSH terms included "ovarian hyperstimulation syndrome," "GnRH agonist," and "ovulation induction." All abstracts and full texts were screened.

Informed consent was obtained from the patients for the publication of these case reports, and ethical guidelines were adhered to throughout the study. The three cases were collected from two different IVF units. The patients were admitted and treated in different gynecological departments for OHSS.

## Case 1

### Clinical fertility history

A 33-year-old nulliparous patient presented at the gynecologic emergency department complaining of abdominal distension, pain, and dyspnea one day after the retrieval of 34 oocytes as part of social fertility preservation. The stimulation cycle was a GnRH antagonist protocol, with recombinant FSH (rFSH), Gonal-F 225 IU (international units) (Merck serono), started on the second day of the menstrual cycle, together with a selective aromatase inhibitor (SAI), letrozole (TEVA^®^) 5 mg. The choice to administer an SAI was taken by her treating physician, trying to minimize hormonal changes that might have an impact on her background autoimmune disease. A GnRH antagonist (Ganirelix 0.25 mg, MSD) was administered from day seven of stimulation. Low molecular weight heparin (enoxaparin, 40 mg) was administered from day eight of stimulation. The stimulation lasted for 12 days, during which the rFSH dose remained unchanged, and the patient received a total dose of 2025 IU of rFSH. Final follicular maturation was triggered with 0.2 mg of GnRH-a (Decapeptyl, Triptorelin, Ferring Global, 0.2 mg). The estradiol concentration on the day of the trigger was 6120 pg/mL (normal range for monofollicular ovulation, estradiol 140–370 pg/mL). No OHSS preventive medication such as cabergoline was administered as the OHSS risk following a GnRH-a trigger was thought to be low.

The patient’s past medical history consisted of PCOS, hirsutism, impaired fasting glucose (IFG), and psoriasis with joint involvement. She had received treatment with methotrexate (MTX) for her psoriasis, which was discontinued four months before the stimulation process. The basal ultrasound showed polycystic ovary-like ovaries. The patient weighed 71 kg with a body mass index of 26.1 kg/m^2^.

### Course

Physical examination in the emergency room revealed abdominal distention and tenderness. Initial lab results showed hemoconcentration: hematocrit 47% (normal range 35–45%), hemoglobin (Hgb) 16.1 gr/dL (normal range 12–16 gr/dL), platelet (PLT) count 461 10^3^/uL (normal range 150–450 10^3^/uL), and leukocytosis -white blood cell count (WBC) 23.1 10^3^/uL (normal range 4–10 10^3^/uL). Electrolytes and kidney function were within normal range. On ultrasound (US), moderately enlarged ovaries (each measured eight cm in diameter) and severe ascites (accumulation of fluid at the level of her liver) were seen. As per department protocol, the patient was admitted for supportive care, including saline (Hartman solution, Baxter, 1500CC per day) and oncotic infusions (Voluven, Hospira, 500CC per day), dopamine agonist (cabergoline 0.5 mg per day), and low molecular weight heparin (enoxaparin, 40 mg per day). Her symptoms and body weight (to monitor fluid management) were followed daily.

On the second day of her admission, the weight of the patient increased by 6.1 kg (78.1 kg), and due to dyspnea and tachypnea along with an oxygen saturation level of 94% on room air, a computed tomography angiography (CTA) scan was performed to rule out pulmonary embolism (PE). The scan revealed the presence of pleural effusions and ascites with no PE. Due to increased patient discomfort, dyspnea, and oliguria, paracentesis only was performed, and 2200 mL of transudate was removed. With continued supportive care, the patient gradually improved, and seven days later, she was discharged in 72.2 kg for follow-up in an outpatient clinic setting.

22 days after oocyte pick-up (OPU), the patient’s physical examination revealed no remarkable findings. There were no signs of abdominal swelling or tenderness. A pelvic ultrasound scan showed that the patient’s ovaries had returned to their normal sizes, and there was no free fluid in the pelvis. Laboratory findings also returned to normal ranges.

## Case 2

### Clinical fertility history

A 22-year-old nulliparous patient presented at the gynecologic emergency department complaining of abdominal distension, pain, and dyspnea a day after the retrieval of 69 oocytes as part of fertility preservation for pre-implantation genetic testing for monogenetic disorders (PGT-M) due to FAP (Familial adenomatous polyposis), with a history of colon cancer. Her ultimate goal is to pursue surrogacy.

The stimulation cycle included rFSH (Gonal-F, Merck serono) 150 IU/day from day two of the menstrual cycle and decreased to 100 IU/day on day 11. SAI (letrozole 5 mg, TEVA^®^) was started simultaneously with rFSH, and a GnRH antagonist (Ganirelix 0.25 mg, MSD) was administered from day six of stimulation. The stimulation lasted 12 days, and the patient received a total dose of 1700 IU of rFSH. Final follicular maturation was triggered with GnRH-a (Decapeptyl, Triptorelin, Ferring Global 0.2 mg). The estradiol concentration on the day of trigger was 3130 pg/mL (normal range for monofollicular ovulation estradiol 140–370 pg/mL). No OHSS preventive medication such as cabergoline was administered as the OHSS risk following a GnRH-a trigger was thought to be low.

The patient past medical history consisted of FAP and a previous history of hemicolectomy due to desmoid tumor. The desmoid tumor has been identified in three specific locations, and it has been stable and effectively treated.

The patient reported regular menstrual cycles and no hyperandrogenism. Her body mass index was 23.7 kg/m^2^ (body weight, 60 kg). The basal US showed polycystic ovary-like ovaries with more than 50 antral follicles.

### Course

Physical examination of the patient exhibited tense ascites and tachypnea. Lab results (normal range referenced in Case 1) showed hemoconcentration (hematocrit 45.8%, Hgb 16 gr/dL), platelet count 230 10^3^/uL, WBC 20.7 10^3^/uL, electrolytes disturbances (hyponatremia of 133 nmol/L and hypokalemia of 2.8 nmol/L). Her creatinine (Cr) and blood urea nitrogen (BUN) levels were within normal range. A US and CT scans revealed enlarged ovaries of 13.7 and 11.5 cm in diameter and ascites expanding to the hepatic region. The patient was treated with saline (Hartman solution, Baxter, 1500CC per day) and oncotic infusions (Voluven, Hospira, up to 500CC per day), dopamine agonist (cabergoline 0.5 mg per day), SAI letrozole 5 mg (TEVA^®^), GnRH antagonist (0.25 mg Ganirelix, MSD), and low molecular weight heparin (enoxaparin, 40 mg per day). She was dyspneic with normal oxygen saturation at room air and oliguria. A day following her admission, the abdominal discomfort was more pronounced, and urine output decreased; a US scan revealed an increase in ascites but no change in ovarian size. Paracentesis only was performed, and 1100 mL of transudate was removed. The following day, a repeat paracentesis was performed with the removal of an additional 1900 mL of transudate. During her hospitalization, and with supportive care, the patient gradually improved and was discharged eight days later for follow-up in an outpatient clinic setting.

20 days after oocyte pick-up (OPU), the patient’s physical examination revealed no remarkable findings. There were no signs of abdominal swelling or tenderness. A pelvic ultrasound scan showed that the patient’s ovaries had returned to their normal size, and there was no free fluid in the pelvis. Laboratory findings, also, returned to normal ranges.

## Case 3

### Clinical fertility history

A 41-year-old nulliparous patient presented at the gynecologic emergency department complaining of abdominal distension, pain, and dyspnea three days following the retrieval of 62 oocytes as part of social fertility preservation. The stimulation cycle was a GnRH antagonist protocol, with recombinant FSH and LH (rFSH + rLH), Pergoveris 300 IU (Merck serono), started on the second day of the menstrual cycle and a GnRH antagonist (Ganirelix 0.25 mg, MSD) administered from day four of stimulation. The stimulation lasted eight days, with three days of Pergoveris 300 IU, tapered down to 225 IU for another three days, and one day of coasting with no gonadotrophin injection. The patient received a total dose of 1575 IU. Final follicular maturation was triggered with 0.2 mg of GnRH-a (Decapeptyl, Triptorelin, Ferring Global, 0.2 mg). The estradiol concentration on the trigger day was 9580 pg/mL (normal range for monofollicular ovulation, estradiol 140–370 pg/mL). No OHSS preventive medication such as cabergoline was administered as the OHSS risk following a GnRH-a trigger was thought to be low.

The patient’s past medical history consisted of depressive disorder. She had received selective serotonin receptor inhibitor (SSRI), Escitalopram 10 mg (Lundbeck), and olanzapine (Zyprexa, 10 mg, TEVA^®^). The basal ultrasound showed polycystic ovary-like ovaries. The patient had a normal early follicular-phase endocrine profile of FSH 4.9 mIU/mL (normal range 2–10 mIU/mL), LH 4.8 mIU/mL (normal range 2–12.5 mIU/mL), E2 38 pgl/mL (normal range 13–42 pg/mL). The patient weighed 60 kg with a body mass index of 22.6 kg/m^2^.

### Course

Physical examination in the emergency room revealed abdominal distension and tenderness. Initial lab results (normal range referenced in Case 1) showed hemoconcentration: hematocrit 40.6%, Hgb 13 gr/dL, PLT count 390 10^3^/uL, and WBC 17.6 10^3^/uL hyperkalemia 5.1 mg/dl, whereas kidney function was within normal range. In the US, enlarged ovaries (each measuring 10 cm in diameter) and severe ascites (fluid accumulation at the level of her liver) were seen. Following department protocol, the patient was admitted for supportive care, including intravenous saline (normal saline 0.9%, Baxter, 2000CC per day) and fluid balance, along with treatment using low molecular weight heparin (enoxaparin, 60 mg per day). Her symptoms and body weight (to monitor fluid management) were followed daily.

Over nine days of admission, the patient’s weight increased by 8 kg (total weight 68 kg). On the fourth day of her admission, due to worsening dyspnea, a chest X-ray was performed, revealing significant pleural effusions. Due to increased patient discomfort, dyspnea, and tachypnea, paracentesis was performed, resulting in the removal of 1500 mL of transudate. With continued supportive care, the patient’s condition was gradually improved, and she was discharged 62 kg five days later for further follow-up in an outpatient clinic setting.

21 days after OPU, a subsequent physical examination of the patient revealed no remarkable findings, with no signs of abdominal swelling or tenderness.

### Discussion

OHSS is a complication of IVF that can lead to hospitalization and, in severe cases, may result in patient mortality. A common approach to reducing the risk of early OHSS is using GnRH-a as a trigger for final oocyte maturation [13], combined with a freeze-all strategy (mitigating the risk of late-onset OHSS( [17, 20, 33, 34]. The suggested mechanisms for the reduced risk associated with GnRH-a trigger compared to hCG can be categorized into two:

1. GnRH-a has a shorter half-life and weaker receptor affinity [35, 36]. Compared to the natural LH surge, which typically lasts 36–48 h, or the hCG trigger with a half-life of up to six days, the GnRH-a induced LH surge lasts only 24–36 h [12].
2. GnRH-a and HCG initiate different downstream receptor cascades: GnRH-a induces luteolysis, resulting in lower levels of steroid hormones [36–39], and is associated with lower levels of vascular endothelial growth factor (VEGF) [37].

Indeed, the implementation of the GnRH-a trigger in combination with the freeze-all approach strategy effectively reduces the risk of severe OHSS [20, 40]. In a retrospective study [10] that reviewed four randomized controlled trials involving high-responder women treated with GnRH-a and the freeze-all strategy, the incidence of OHSS was examined. Among a total of 77 women included in the study, 22% developed mild OHSS, characterized by signs and symptoms lasting between 6 and 21 days. Importantly, no cases of severe OHSS were observed, and none of the reported OHSS cases were classified as serious adverse events. These findings suggest that while the incidence of mild OHSS may occur in a subset of high-responder women, the occurrence of severe OHSS is rare [40]. However, there are still cases where women develop OHSS despite following this protocol.

To date, 10 cases (Table 1) have been reported documenting severe early OHSS following GnRH-a trigger. In five cases, these individuals experienced severe symptoms within the first 12–36 h following oocyte pick-up (OPU), and in five cases, symptoms appeared 4–7 days post-OPU. It is noteworthy that in all of the reported cases of early severe OHSS, more than 25 oocytes were aspirated during the procedure [33, 34, 41, 42].

**Table 1:** Papers Reporting OHSS following GnRH-a Triggering only

Name	Year	Number	Ovulation trigger	Num. of oocytes	Luteal Support	Timing	Embryo transfer	OHSS
Georg Griesinger et al. (2011)[33]	2011	*3/51	Triptorelin 0.2 mg	36	Not mentioned	72 h post-OPU	No	Enlarged ovaries/Mild *Only one reported
		1/51	Triptorelin 0.2 mg	13	No	12 h post-OPU	No	Severe, diagnosed as hemoperitoneum
Ayse Seyhan et al. (2013) [25]	2013	2/5	GnRH-a trigger	23–26	No	4–6 days post-OPU	Yes	Severe
Ali Sami Gurbuz et al. (2014)[34]	2014	3	Leuprolide 1 mg	27–42	Not mentioned	12–48 h post-OPU	No	Severe
Liza Ping Ling et al. (2014)[42]	2014	1	Triptorelin 0.2 mg	41	Not mentioned	12 h post-OPU	No	Severe
Human Mousavi Fatemi (2014)[41]	2014	2	Triptorelin 0.3 mg Triptorelin 0.2 mg	30	Not mentioned	24-h post-OPU 6 days post-OPU	No	Mild/Severe
Samuel Santos-Ribeiro (2015) [26]	2015	1	Triptorelin 0.2 mg	59	No	12 h post-OPU	No	Mild
Shevach Friedler et al. (2019) [27]	2018	1	Triptorelin 0.2 mg	16	Nafarelin acetate 400 lg/day	6 days post-OPU	Yes, Blastocyst	Moderate
Neeta Singh et al. (2020) [31]	2020	1	Leuprolide 2 mg	11	Not mentioned	72 h post-OPU	No	Moderate
Lorio et al. (2021) [29]	2021	1	Triptorelin 0.2 mg	16	No	7 days post-OPU	No	Severe
M Fernández-Sánchez et al. (2023)[10]	2023	17/77	Triptorelin 0.2 mg intranasal buserelin 600 μg nafarelin 800 μg	10–41	Yes, not in all patients	6–21 days post-OPU Early < 9 days	Yes, not in all patients	Mild
Nazanin Hajizadeh [30] (2023)	2023	1	Variopeptyle 0.3 mg	21	Cabergoline 0.5 mg Cetrotide 0.25 mg	6 days post-OPU	No	Severe

What predisposes a certain individual to develop severe early-onset OHSS despite using the GnRH-a trigger and freeze all deserves further study. Still, a possible explanation could be related to the function of the Follicule-stimulating hormone receptor (FSH-r). Specific mutations and genetic predispositions related to the FSH-r have been identified as an etiological factor for spontaneous OHSS in several studies [12–15, 33]. These mutations have been associated with recurrent OHSS occurring during normal menstrual cycles or natural pregnancies without assisted reproductive treatments. There have been cases of familial OHSS, suggesting a genetic component to the condition [43]. It is plausible that polymorphism in the FSH-r may be responsible for some of the severe OHSS cases following the GnRH-a trigger. While genotyping the FSH-r or luteinizing hormone receptor (LH-r) of each patient to identify polymorphisms may provide insights into the occurrence of severe OHSS following a GnRH-a trigger and freeze-all protocol, it may not be cost effective due to the complexity and rarity of such cases.

A common feature in the case reports we described is the reliance on GnRH-a as the sole factor for OHSS prevention. Gonadotropin doses were not reduced during stimulation, and prophylactic medication such as Cabergoline was not used. The ASRM guidelines [44] describe risk factors before, during, and after stimulation that put a patient at risk for developing OHSS. These risk factors include the patient’s ovarian reserve, the number of growing follicles, the estradiol levels during stimulation, and the number of oocytes retrieved. Careful consideration should be given to high-response patients regarding ovulation-stimulating dosages and prophylactic actions rather than relying solely on the agonist trigger to reduce the risk.

## Conclusion

In this study, we reported on three cases of severe early-onset OHSS following the administration of a GnRH-a trigger along with a freeze-all strategy. Despite these preventive measures, all cases required hospitalization. While these strategies have been effective in reducing the risk of OHSS, some cases still occur. Genetic factors, particularly FSH-r mutations, may play a role in these occurrences. By conducting an extensive literature review, we identified 10 previously documented cases with similar characteristics, indicating the rarity of this phenomenon. Nevertheless, clinicians must be aware of this potential complication and its associated severity when it does occur. Further research and larger studies are warranted to accurately determine the incidence of severe early OHSS following GnRH-a trigger.
