# Correction: Severe ovarian hyperstimulation syndrome following sole gonadotropin-releasing hormone (GnRH) agonist trigger: a case series and literature review

**DOI:** 10.1007/s00404-026-08412-4

**Published:** 2026-04-08

**Authors:** Roza Berkovitz-Shperling, Nivin Samara, Reut Meir, Omri Dominsky, Foad Azem, Ido Feferkorn

**Affiliations:** 1 Department of Obstetrics and Gynecology, Lis Hospital for Women’s Health, Tel Aviv. Sourasky Medical Center, affiliated to the Faculty of Medicine, Tel Aviv University, 6423906 Tel Aviv, Israel; 2 Department of Obstetrics and Gynecology, Shaare Zedek Medical Center, 6 Weizman St., Affiliated to Faculty of Medicine, The Hebrew University of Jerusalem, Jerusalem, Israel

**Correction: Archives of Gynecology and Obstetrics (2024) 310:2297–2304** 10.1007/s00404-024-07740-7

In this article the author’s name Foad Azem was incorrectly written as Foad Azam. The original article has been corrected.

